# The Early Stages of Heart Development: Insights from Chicken Embryos

**DOI:** 10.3390/jcdd3020012

**Published:** 2016-04-05

**Authors:** Johannes G. Wittig, Andrea Münsterberg

**Affiliations:** School of Biological Sciences, University of East Anglia, Norwich Research Park, Norwich NR4 7TJ, UK; j.wittig@uea.ac.uk

**Keywords:** chick embryo, fate mapping, heart fields, morphogenesis, *in ovo* studies

## Abstract

The heart is the first functioning organ in the developing embryo and a detailed understanding of the molecular and cellular mechanisms involved in its formation provides insights into congenital malformations affecting its function and therefore the survival of the organism. Because many developmental mechanisms are highly conserved, it is possible to extrapolate from observations made in invertebrate and vertebrate model organisms to humans. This review will highlight the contributions made through studying heart development in avian embryos, particularly the chicken. The major advantage of chick embryos is their accessibility for surgical manipulation and functional interference approaches, both gain- and loss-of-function. In addition to experiments performed *in ovo*, the dissection of tissues for *ex vivo* culture, genomic, or biochemical approaches is straightforward. Furthermore, embryos can be cultured for time-lapse imaging, which enables tracking of fluorescently labeled cells and detailed analysis of tissue morphogenesis. Owing to these features, investigations in chick embryos have led to important discoveries, often complementing genetic studies in mice and zebrafish. As well as including some historical aspects, we cover here some of the crucial advances made in understanding early heart development using the chicken model.

## 1. Introduction

The detailed mechanistic understanding of developmental processes is a major requirement to be able to identify the embryonic origin of diseases and to develop future therapeutic interventions. Different model organisms have been established to study patterning and organogenesis in developing embryos. Important metazoan model organisms include the nematode (*Caenorhabditis elegans*), the fruit fly (*Drosophila melanogaster*), the tunicate (*Ciona intestinalis*), a few species of sea urchin, the teleost fish (*Danio rerio*), the African claw-toed frog (*Xenopus laevis*), the mouse (*Mus musculus*), and the chicken (*Gallus gallus*). All of these have different advantages and have made significant contributions to our understanding of developmental processes. The focus of this review will be the chicken, specifically its role in our current understanding of early heart formation.

The chicken is a “classic” model organism and the first meaningful information obtained through its use arose in the 17th century, when it was shown that embryos are not preformed but develop body parts progressively. Further fundamental discoveries were dependent on the development of optical microscopes, which made it possible to discover the three germ layers: ectoderm, mesoderm, and endoderm. A comment on Charles Bonnet’s ideas on “fecundation” and development of the germ (egg) was published in the late 19th century [1]. Since then developmental biology research has changed dramatically owing to advances in genetics and in cell and molecular biology, which enabled much progress and a “golden age” for the discipline [2]. Analyses have become more sophisticated, focusing on discrete regions in the developing animal.

The chick embryo is ideal for studying the early development of the heart, the first functioning organ in the embryo. A major advantage is that the chick develops *ex utero* in an egg, which allows easy accessibility during all stages of development post-laying. This ease of access enables *in ovo* manipulations and observation of the embryo, such as dissection, grafting, micro-injection, and labeling, and this has made the chicken popular, even before the molecular age [3,4,5]. Particularly powerful have been grafting and ablation experiments. When combined with the use of quail/chick chimeras [6], this approach allowed the tracing of grafted cells before genetic labeling became possible. Establishing methods for *ex ovo* development and introduction of constructs encoding fluorescently labeled proteins by electroporation has facilitated the imaging of cell movement in live embryos using advanced microscopy [7,8]. Advanced tools for image registration allow for the alignment and comparison of multiple specimens in the absence of morphological landmarks [9]. By directly labeling the extracellular matrix, it has also been possible to measure active *versus* passive motion of cells, including cardiac progenitors, during gastrulation [10,11]. The use of CRISPR/Cas9-mediated genome editing via targeted electroporation allows the generation of genetic mosaics; combined with imaging the behavior of mutant cells can then be studied in detail, for example in developing somites [12]. Furthermore, improved methods for transgenesis and the availability of lines, both quail and chick, transgenic for fluorescent markers expressed either ubiquitously or restricted to specific cell lineages, has enhanced the utility of avian models [13,14,15].

Finally, the mature chick heart comprises four chambers with in- and out-flow tracts, and despite some differences, for example during septation and aortic arch remodeling [16], it resembles the human anatomy more closely than other non-mammalian model organisms. Owing to those features, and the available tool-kit described above, avian embryos will almost certainly continue to contribute significant insights into the development of the heart.

## 2. Cardiac Development and Morphogenesis

### 2.1. Mapping Studies and Characterization of Cardiogenic Fields

In the chick embryo, systematic observations and comparative analyses were boosted when Hamburger and Hamilton established a classification scheme for developmental stages that was universally adopted [17]. A recent reference guide maps the stages of heart development onto the HH-stage series [18]. In addition, the series has been refined for the stages of gastrulation [19], which starts with the formation of the primitive streak in the midline of the embryo.

In the early chick gastrula (Hamburger-Hamilton, HH stage 3), cardiac progenitors are located in the mid-primitive streak, from which they ingress to enter the mesoderm bilaterally [20,21,22,23]. By HH4, the late gastrula/early neurula stage, the contribution of the primitive streak to the heart ceases [21,24]. At that stage precardiac areas are organized into bilateral heart fields located in the lateral plate mesoderm, which subsequently splits into the somatic and splanchnic layers, the latter comprising cardiogenic cells. Bilateral heart fields were originally characterized by culturing isolated cells and testing their potential to generate spontaneously contracting cardiomyocytes [20,25].

Early studies tracing cardiac cells in gastrula stage embryos used isotope labeling and autoradiography, thus defining bilateral heart fields that are initially separate but then fuse to generate the tubular heart at early somite stages [26]. In mouse embryos, the timing is different and the heart field mesoderm merges together across the midline at the 1-somite stage (E7.5), forming a “crescent” [27,28].

Additional insights regarding the origin of cells contributing to the heart as well as the aortic arches derived arteries were obtained through interspecies grafts that generate quail–chick chimeras. This approach, developed by Lièvre and Le Douarin [29], was important for studies in avian model systems and a reliable and sensitive alternative to methods involving radioactive isotopes [25]. Using quail–chick chimeras and fluorescent vital dye injections, a more precise fate map was generated [21]. This showed that cardiomyocyte and endocardial precursors arise from a rostral portion of the HH3 primitive streak, and that the craniocaudal organization of cells within the streak reflects the craniocaudal arrangement of the linear heart tube [21], extending the earlier cardiogenic “potency map” of the primitive streak by DeHaan [20]. The linear heart tube becomes extended and refined by additional cell populations contributing to the mature heart (see Section 2.3).

### 2.2. Pre-Gastrula and Gastrula Stages

#### 2.2.1. Specification and Migration of Cardiac Progenitor Cells

Cardiogenic potential can be detected in pre-streak, blastula stage embryos prior to gastrulation before the heart fields emerge. Pre-streak stage chick embryos are a flat disc composed of two layers, the epiblast (upper layer) and the hypoblast (lower layer). Cardiac progenitors are found within the posterior half of the epiblast [30] and these cells have cardiogenic potential in culture [31,32]. These authors also showed that the hypoblast is required to induce cardiac myogenesis in the early epiblast, and furthermore, that Tgfβ/activin is sufficient to substitute for its cardiogenic-inducing ability [31,32]. In contrast, BMP-2 and BMP-4 inhibit cardiogenesis at this stage, consistent with studies that show that BMP antagonists, such as chordin, can induce the expression of the early marker, smooth-muscle alpha actin (SMA), in cultured posterior epiblasts at pre-gastrula stages [33]. In mice, transplantation experiments combined with embryo culture showed that epiblast cells can acquire a cardiac fate independent of ingression through the primitive streak [34]. Thus, in both chicks and mice, ingression itself is not necessary for fate specification.

Soon after gastrulation, prospective cardiac cells migrate to the anterior lateral mesoderm and the bilateral heart fields contain prospective endocardial and myocardial cells, indicating that cardiac fates are allocated in the primitive streak or earlier prior to cell migration. This idea was confirmed using lineage tracing with low titers of a replication-defective retrovirus expressing LacZ. The labeled cells gave rise to either myocardial or endocardial derivatives [35].

Using chick embryos and *ex vivo* tissue recombination experiments it was possible to identify the origin of signals in the endoderm underlying the bilateral heart field mesoderm in the anterior lateral plate that trigger the commitment to the cardiac lineage [36]. Pioneering studies identified the crucial role of BMP signaling post-gastrulation. Beads soaked in recombinant BMP-2 could induce ectopic expression of early cardiac markers, such as the transcription factors GATA-4 and Nkx-2.5. Furthermore, recombinant BMP-2 or BMP-4 protein induced myocardial differentiation and beating in explants of non-cardiogenic mesoderm, while exposure to the secreted protein Noggin, a BMP-antagonist, completely inhibited differentiation of precardiac mesoderm [37,38]. The competency to respond to BMP-2/4 alone was stage dependent [39] and restricted to anterior mesoderm explants. Subsequently it was shown that interactions between BMP-2 and FGF-4 pathways are important for the induction of cardiac cell fate in the posterior mesoderm [40] by directly targeting the transcription factor Nkx2.5 [41].

Additional experiments conducted in both chick and Xenopus gastrula stage embryos revealed that inhibition of canonical Wnt/β-catenin signaling is critical for heart development [42,43], whereas β-catenin-dependent Wnt signaling in the posterior lateral mesoderm induced hematopoiesis [42]. The Wnt family of secreted proteins initiates several signal transduction pathways, recently reviewed in the context of heart development [44]. Antagonists of β-catenin-dependent Wnt signaling that promote cardiogenesis include dickkopf (Dkk1) and crescent. In chicks, crescent is expressed in anterior endoderm during gastrulation and can induce the expression of cardiac genes in posterior, non-cardiogenic tissues *in vitro* [42]. The conditional genetic ablation of β-catenin in early mouse embryos also led to a proposed cell fate switch and ectopic heart formation [45]. These observations are consistent with the idea that β-catenin-dependent Wnt signaling represses cardiogenesis; however, this is context dependent. At an early stage of development, prospective cardiac cells are exposed to canonical Wnt-ligands: both Wnt-3a and Wnt-8c (known as Wnt8a in mouse and human) are expressed in the primitive streak. Indeed, during the differentiation of embryonic stem cell derived embryoid bodies, Wnt/β-catenin signaling is initially required for induction of mesoderm and thus cardiomyogenesis. Therefore, this pathway either enhances or inhibits cardiogenic differentiation depending on the stage of development; it has been proposed that canonical signaling retains cardiac precursors in a proliferative precursor state, whereas non-canonical signaling promotes their differentiation (reviewed in [44,46]).

Taken together, work in avian embryos demonstrated that inhibitors of β-catenin-dependent Wnt signaling act in concert with BMP and FGF signaling molecules to specify cells to cardiac fates during early neurula stages. These insights led to efforts to differentiate human pluripotent stem cells into cardiomyocytes [47]. Additional data indicate that FGF and BMP signaling pathway interactions are regulated by negative feedback loops involving microRNAs, particularly miR-130 and miR-133 [48,49].

Furthermore, β-catenin-independent (or non-canonical) signaling is important for cardiogenesis. Wnt binding to frizzled receptors and signaling through Dvl can activate alternative pathways, including the planar cell polarity (PCP) and Wnt/Ca^2+^ pathways [44]. Known mediators of the Wnt/PCP pathway involve the ligand Wnt-11 and the small GTPase RhoA. In chicken embryos, RhoA controls tissue polarity and cell movement of cardiogenic progenitors [50,51]. Live-imaging and cell tracking of cardiac progenitors have shown that during gastrulation a combination of BMP-2/4- and Wnt/GSK3β-mediated signals is involved in controlling the migration of these cells towards the bilateral heart fields [52]. This work also showed that the two pathways are integrated by differential phosphorylation of Smad-1: (1) at the carboxy-terminus in response to BMP-receptor activation; and (2) in the linker region by GSK3β kinase.

**Figure 1:**
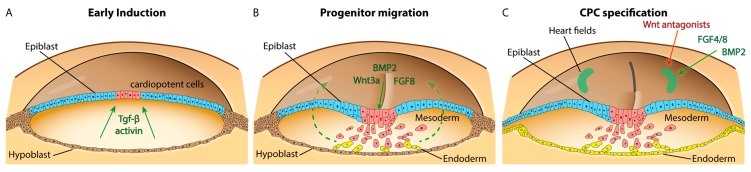
Cardiogenic signals in pre-gastrula, gastrula, and neurula stage embryos. Schematic representation of a pre-gastrula chick embryo (**A**) with epiblast and hypoblast layers. Cardiopotent cells identified in the posterior epiblast respond to Tgf-β/activin signaling. The diagram in (**B**) represents a HH3 gastrula with prospective mesoderm (**red**) and endoderm cells (**yellow**) ingressing through the primitive streak. Wnt3a, BMP2, and FGF8 are expressed in the primitive streak and control migration trajectories of cardiac progenitor cells, indicated by **green** stippled arrows, towards the bilateral heart fields. (**C**) Representation of a neurula stage embryo, approximately HH5. Gastrulation continues at the primitive streak, which is regressing; an endoderm layer has formed, and cardiogenic cells are located in bilateral heart fields in the anterior lateral plate mesoderm. A combination of BMP2, FGF4/8, and inhibitors of canonical Wnt signaling act to specify cardiac fate.

These observations suggest that the control of migration is intimately linked with that of cell fate specification—the same players and pathways are involved in both processes and this is illustrated in Figure 1. However, the downstream effectors and molecular switches that control the cells’ response depending on their competency and differentiation status remain to be identified.

Effects of BMPs on progenitor cell migration in addition to effects on fate acquisition are also consistent with observations in genetically altered mice. For example, the conditional deletion of BMP receptor type 1a using mesoderm-posterior-1-Cre (MesP1-cre), which acts in cardiogenic progenitors, results in the absence of the entire cardiac crescent and the restricted expression of myocardial progenitor markers Nkx2-5 and the LIM homeobox 1 transcription factor, Isl1, to a small remaining cardiac field [53]. Consistent with the findings in chick embryos, these authors also showed that sustained activation of canonical Wnt signaling led to increased Isl1 expression but inhibited heart tube formation at the eight-somite stage [50,53]. Thus far it has not been possible to observe cardiac progenitor cell migration in real time using mice; however, advanced imaging approaches will soon be able to address this challenge [54].

#### 2.2.2. Establishment of Left–Right Asymmetry

Shortly after the emergence of cardiogenic progenitors from the primitive streak and around the time that they arrive in the heart fields, the bilateral symmetry of the early embryo is broken. Ultimately this leads to the striking left–right asymmetry in the placement and differentiation of organs, which is seen in all vertebrates. Experiments in chick embryos have made major contributions to our understanding of the mechanisms involved in this process. For a review see [55]. In particular, the gene network that provides left–right information was characterized in chick embryos [56]. Initial breaking of symmetry starts at Hensen’s node, the organizing center at the anterior end of the fully extended HH4 primitive streak. Several signaling molecules are asymmetrically expressed, including activin receptor IIa, Sonic hedgehog (Shh), and cNR1 (the chick homologue of mouse nodal); the experimental manipulation of these pathways, through implantation of growth factor soaked beads or cell pellets, affects heart *situs* [56]. Furthermore, recent work showed that *N*-cadherin is involved in asymmetric gene expression and the leftward cell movements in Hensen’s node [57].

In mice, the use of a nodal-lacZ reporter allele confirmed its asymmetric expression on the left side [58]. Although the mechanisms leading to initial breaking of symmetry are different in mice and chicks [59,60], in both species the transcription factor Pitx2 acts downstream of nodal and Shh signaling. In chick embryos misexpression of Pitx2 is sufficient to produce reversed heart looping [61]. The literature on genetic manipulations of Pitx2 is extensive and cannot be covered here; suffice it to say that cardiac laterality defects are usually observed (for example [62], and references in [55]).

The signaling molecules expressed on the left side interact with a right-sided program, initiated by BMP-4 at Hensen’s node inducing FGF8, which in turn activates Snai1, a Zn-finger transcriptional repressor. Snai1 is necessary for the formation of the proepicardium (PE), which in the chick develops only on the right side—a vestigial PE on the left undergoes apoptosis. Ectopic expression of FGF8 or Snail on the left led to bilateral PE formation [63]. In the mouse, the PE, which is characterized by expression of WT1 and TBX18, develops bilaterally. This may reflect differences in FGF8, which is a determinant of the right side in the chick but mediates left side identity in mice [59,64].

### 2.3. Discovery of Additional Heart Fields

**Figure 2:**
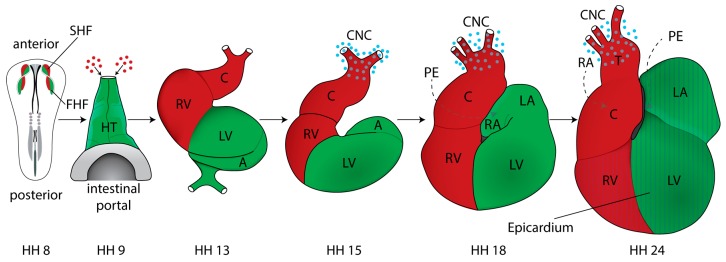
Cardiac morphogenesis in chick embryos. Schematic ventral views of HH8 to HH24 chick hearts. Fate mapping revealed the location of first and second heart fields (FHF, SHF), marked in green and red. Fusion generates a primitive heart tube by HH9; secondarily added cell populations have not yet entered (red dots). In all images, components of the heart derived predominantly from FHF are in green and components derived predominantly from SHF and also AHF are in red. During dextral-looping the straight heart tube transforms into a C-shaped bend by HH13 and SHF/AHF-derived cells contribute to the heart; primitive atria move dorsocranially. Further positional changes are indicated. The proepicardium (PE) is located on the dorsal side (stippled grey arrow); it generates the epicardium. The expansion of the epicardium over the heart by HH24 is indicated by stripes. The cardiac neural crest (CNC), shown as blue spots, contributes to outflow tract septation and remodeling of the great arteries. See text for details. A, atrium; C, conus, CNC, cardiac neural crest; HT heart tube; LA/RA, left/right atrium; LV/RV, left/right ventricle; T, truncus arteriosus.

Classic mapping experiments using labeling with iron oxide particles followed by time-lapse photography indicated that new segments are added to the linear heart tube during looping, in particular to generate outflow myocardium [65,66]. Cells residing in the ventral region of the subcephalic fold of HH9^−^ were shown to be included at the cephalic end of the heart tube by HH12. Similar labeling showed that precursors for the right and left primitive atria are not yet present in the HH8–9 straight heart tube [67] but become incorporated later during loop stages. Building on this early work, the origins of secondarily added cell populations were characterized in more detail in both the chick and mouse, using fluorescent dye or genetic labeling, respectively [68,69,70]. This showed that cell populations contributing to the outflow tract (OFT) are located in the pharyngeal mesoderm and the splanchnic mesoderm anterior, and immediately adjacent to the straight heart tube. These regions have been termed the anterior and secondary heart fields (AHF/SHF), respectively, and their derivatives are shown in Figure 2. The cells contributing to the OFT express the transcription factors Nkx2.5 and GATA-4. They are also positive for HNK-1 immunostaining as they translocate into the heart [69,70]. Using vital dye injections and tissue grafting it was possible to map the location and ingression sites of prospective AHF and SHF cells in the primitive streak of gastrula stage HH3 chick embryos [71]. This work showed that during early somite stages the Isl1-positive AHF progenitors were located in the cranial paraxial mesoderm and the pharyngeal mesoderm [71], also consistent with studies that identified a close relationship between these progenitors and some craniofacial skeletal muscles, in both the chick and mouse [72,73].

*In vivo* live imaging in quail embryos was used to determine the origins of the endocardium. This identified an endocardium-forming field located medial to and distinct from the first and second heart fields. These progenitors are restricted in their potential and enter the heart from the arterial pole [74]. Conditional genetic ablations showed that in the mouse the origins of the endocardium are more heterogeneous [74,75] and are specified by a gene network initiated by the early cardiac transcription factor Nkx2.5 [76].

In the mouse, cells that generate in particular the right ventricle and outflow myocardium were characterized through the expression of an FGF-10 lacZ knock-in allele in the pharyngeal mesoderm [68]. The second heart field populations of cells are reviewed in detail in [77,78]. Additional makers have since been identified and genetic studies in mice have helped to explain congenital heart defects that affect the OFT, comprising the aortic and pulmonary trunk [79]. OFT septation and the remodeling of the great arteries also depend on the neural crest (see below), which adds to the complexity of some mutant phenotypes.

Work in chick embryos investigating a signaling mechanism within the AHF niche showed that BMP and FGF crosstalk coordinates the balance between proliferation and differentiation of cardiac progenitors [80]. Close interaction with cardiac neural crest cells was also shown to be required for the regulation of AHF cell differentiation [81]. Furthermore, studies in both the chick and the mouse have revealed the close relationship between head skeletal muscles and AHF/SHF-derived cardiac muscles, which share overlapping expression of a genetic program that is evolutionarily conserved [73,82,83,84] (reviewed in [85,86]).

More recently the origin of pacemaker cells (PC) of the sinoatrial node (SAN) was identified in a “tertiary” heart field. Using electrophysiological measurements in chick embryos, it was shown that mesoderm cells in a region posterior to the HH8 stage heart fields generate action potentials. By late looping stages these cells contribute PCs of the sinoatrial node. This work also revealed that Wnt8c promotes PC fate [87]. Prior to this, voltage sensitive dyes had been used to monitor spontaneous action potential activity, which was detected at 7–8 somite stages in the pre-beating heart using optical recording [88].

### 2.4. Formation and Transformation of the Straight Heart Tube

Insights regarding the origin of cardiac precursors in pre-gastrula stage embryos and cardiogenic fields at gastrula stages were not among the first investigations into heart formation in the chick. Studies about morphology and how an organ acquires its final form were conducted much earlier. For example, the process of heart looping was first observed in 1758 by Albrecht Haller (cited in [89]), who noticed a transformation of the heart tube into a loop-like shape during heart maturation. Even though it was discovered early, a comprehensive summary of this phenomenon did not appear in the literature until 1922, when the term “cardiac looping” was introduced [90].

Insights into the formation of the heart tube itself included the discovery of the bilateral heart fields, which migrate to the midline and fuse [26]. Initial experiments conducted to analyze the process of fusion determined a craniocaudal course of the merging of the endocardial and myocardial heart primordia [25]. However, this observation was revised to show that fusion occurs in a central region and progresses in cranial and caudal directions, similar to what had been observed in mouse embryos [66].

Our understanding of the molecular and cellular drivers of the fusion process is still limited, but evidence in the chick supports a mechanical role for the endoderm at the anterior intestinal portal. Tracking experiments combined with the use of the myosin-II inhibitor, Blebbistatin, and computational modeling showed that shortening of the endoderm, driven by cytoskeletal contractions, is involved in motion of the heart fields towards the midline [91]. Disruption of the fusion process leads to *cardia bifida*, a severe malformation of the heart, which can be experimentally induced. For example, after surgical incision along the midline of a HH7 chick embryo, two separate contractile tubes form [92]. *Cardia bifida* was also observed in MesP1 null mice, most likely because the migration of mesoderm progenitors was affected [93]. Furthermore, in chick embryos *cardia bifida* was seen after inhibition of the RhoA GTPase, by siRNA, or by electroporating mutant forms of RhoA into cardiac progenitors in the HH3 primitive streak [50,51]. This implicates RhoA-mediated regulation of cytoskeleton dynamics in directional movements of cardiogenic progenitors. The effects of RhoA mutants mimicked what was seen after overexpression of Wnt3a, which controls cardiac progenitor cell migration (see above), potentially through chemotactic guidance [50]. Interestingly, non-canonical Wnt-signaling via Rho GTPase was shown to be important during midline conversion of organ primordia, including heart tube assembly in zebrafish [94]. *Cardia bifida* will lead to embryonic death rather than a congenital heart defect. Nevertheless, mechanistic studies resulting in *cardia bifida* will provide important information about the relative contributions of the primary germ layers and signaling pathways involved in early heart morphogenesis.

After formation of the straight heart tube the looping process begins—reviewed and updated by Männer J. [95]. Major advances made during the late 20th century describe cardiac looping in four phases: (1) the pre-looping phase (HH8–9); (2) the phase of dextral looping, leading to the transformation of the originally straight heart tube into a C-shaped bend/loop whose convexity is directed toward the right of the body (HH9^+^–13); (3) the phase of transformation of the C-shaped heart loop into the S-shaped heart loop (HH14–16); and (4) a phase of late positional changes of the primitive outflow tract (conus) with respect to the atria, with the process being completed by HH24 [95]. For more information about heart looping and a series of pictures, see Figure 2 and the following reviews and books [95,96,97].

Despite the fact that detailed observations and descriptions of heart looping were acquired some time ago, our understanding of the relevant mechanical forces is still in its infancy. Important biomechanical processes include major morphogenetic events such as cranial flexure, which is intimately linked with the caudal shift of the ventricular bend. Some evidence suggests that the bending head and neck regions lead to a compression of the heart loop; however, the converse scenario whereby the caudal shift exerts a pulling force on the head cannot be completely excluded at present [95]. Additional mechanical force is exerted by increased blood flow and blood pressure, and it is evident that altered hemodynamics can contribute to laterality and congenital heart defects [96]. Modern imaging approaches, including light sheet microscopy, which can image live tissues without inducing photo-damage, and computational modeling in combination with studies of cell behavior are key technologies for advancing this field [8,54]. For a summary of approaches for the heart in chicks and other model organisms, see [98].

### 2.5. Cardiac Neural Crest

Experiments using avian embryos, particularly quail–chick chimeras, enabled the analysis of neural crest cell (NCC) migration and differentiation [29,99]. This approach revealed an important contribution by NCCs to the heart. Specifically, replacing chick NCCs arising from the posterior hindbrain adjacent to somites 1–3 with that of quail NCC showed that these cells contribute to the aortico-pulmonary and conotruncal septa; thus they were called “cardiac” NCCs [100,101], although they also contribute to non-cardiac tissues. Cardiac NCCs are crucial for the remodeling of the pharyngeal arteries into an aortic arch, and for septation of the outflow tract into the pulmonary artery and aorta. In mouse embryos, the use of genetic labels such as Wnt1-cre and ROSA26 reporter lines enabled the tracking of cardiac neural crest cell derived tissues [102].

More recently, it has been shown in chick embryos that the chemokine stromal cell-derived factor-1 (SDF1) and its cognate receptor, Cxcr4, are important for the migration of cardiac NCCs towards the heart. This suggested that SDF1 acts as a chemoattractant for cardiac NCCs. Misregulation of SDF1 signaling caused cardiac anomalies including incomplete septation of the aorta and pulmonary trunk (also described as Persistent truncus arteriosus or PTA), and ventricular septal defects (VSD) [103]. The experiments in chicks were consistent with observations demonstrating that mice deficient for Sdf1 or its receptors, Cxcr4 and Cxcr7, exhibit ventricular septal defects [104]. The important role of cardiac NCCs for the etiology of common congenital birth defects, including outflow tract septation defects, has been reviewed (for example, [16]).

### 2.6. Cardiac Chambers

Following heart looping, maturation of the heart into four chambers, two atria and two ventricles, is initiated. The primitive atrium becomes divided by the formation of a septum primum. This septum initiates from the dorsocranial atrial wall at HH14 and grows towards the developing endocardial cushions in the atrioventricular canal (AVC). It has been shown that reciprocal myocardial–endocardial interactions coordinate the formation of valves [105] that optimize blood flow. In addition, qPCR analysis of microRNAs demonstrated distinct expression profiles within the atrial, ventricular, and atrioventricular canal regions of the developing chick heart. In particular miR-23b, miR-199a, and miR-15a displayed increased expression during early AVC development and characterization of target genes suggests that they are involved in regulating epithelial-mesenchymal transition (EMT) signaling pathways [106].

Around the same time, the chamber walls undergo morphological changes. At first, the myocardial layer of the ventricular walls forms protrusions, called trabeculae, which project into the chamber lumen and are covered by a layer of endocardium. The process of trabeculae formation begins at HH16 at the outer curvature of the primitive ventricle—later trabeculae contribute to ventricular septation. Trabeculae grow in length; when growth ceases their shape and morphology change. During this phase of remodeling, trabeculae start to thicken at their anchors in the chamber wall. In the chick, a compact myocardium with a mature trabeculae network is formed around halfway through gestation, by approximately HH34. Throughout embryonic stages the increased surface area generated by trabeculae supports nutrition and oxygen uptake prior to vascularization. Post-birth trabeculae prevent suction, specifically the flow of blood back into the atria. For a more detailed description readers are referred to reviews [107,108] and the references therein.

### 2.7. The Proepicardium

Concomitant with the initiation of trabeculation, cells of the proepicardium migrate to the post-looped heart to form its outermost layer, the epicardium, which invades the myocardial wall, resulting in establishment of the coronary vasculature and an increased number of cardiac fibroblasts in the myocardial wall [109,110,111]. Failed fusion of the proepicardium to the heart results in severe coronary and heart defects and a better understanding of its precise roles will be needed to develop new therapies [112]. Loss-of-PE-function can be induced by photoablation and this induces long-lasting abnormalities in the heart, including a thin myocardium and defects in the coronary vasculature [113]. Interestingly, the epicardium of the distal OFT has a different embryonic origin and gene expression profile, as shown by transplantation and mapping studies [114]. Quail–chick grafting also demonstrated that the PE contributes hemangioblasts but not lymphangioblasts [115]. In both the chick and the mouse. RANKL/NFATC1 signaling induces expression of extracellular matrix-degrading enzymes, which is important for the invasion of epicardial cells into the myocardium [116]. Work in chick embryos examined PE origin [117] and showed that myocardium-derived BMP signals induce the protrusion of Tbx18/WT1-positive proepicardial cells toward the looping heart tube [118]. In both humans and chicks, Tbx5 is implicated in the migration of proepicardial cells [119]. Genetic lineage tracing in mice identified a sub-compartment of proepicardial cells positive for Scleraxis (Scx) and Semaphorin3D (Sema3D), which give rise to coronary vascular endothelium and contribute to the early sinus venosus and cardiac endocardium [120].

## 3. Conclusions

**Figure 3:**
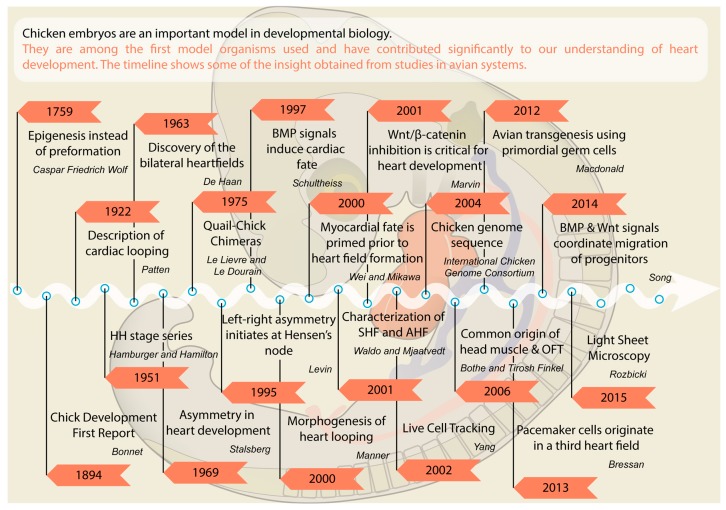
Timeline of important discoveries in chick embryos.

Compared to mammalian model organisms, the chick has discrete advantages for experimental embryology. Due to long generation times, genetic approaches are not straightforward in the chicken; however, *in ovo* accessibility allows transient gain- and loss-of-function approaches, which compensates for this shortfall. In this review we have illustrated how approaches in the chick model have facilitated important insights into the origin of cardiogenic cells and the developmental signals involved in their specification and migration. The timeline in Figure 3 summarizes some crucial milestones. No doubt, ongoing and future work using avian species will provide more original insights into the molecular and cellular mechanisms that underpin the early development of the vertebrate heart.

## Abbreviations

The following abbreviations are used in this manuscript:

AHF/SHF	anterior/secondary heart field
HH	Hamburger-Hamilton
NCC	neural crest cells
OFT	outflow tract
PC	pacemaker cell
PE	proepicardium
